# Social Determinants of Health Discussed with Mothers During Personal Visits Before and During the COVID-19 Pandemic

**DOI:** 10.1089/heq.2020.0140

**Published:** 2021-09-03

**Authors:** Rachel G. Tabak, Cynthia D. Schwarz, Allison Kemner, Shelly Johnston, Adriana Aramburu, Debra Haire-Joshu

**Affiliations:** ^1^ Center for Diabetes Translation Research, Washington University in St. Louis Brown School, St. Louis, Missouri, USA.; ^2^ Parents as Teachers National Center, St. Louis, Missouri, USA.; ^3^ Department of Medicine, Washington University in St. Louis School of Medicine, St. Louis, Missouri, USA.

**Keywords:** COVID-19, social determinants of health, home environment

## Abstract

**Purpose:** This report describes the social determinants of health (SDOH) discussed during personal visits at the time leading up to and during the first 4 months of the pandemic from families across the United States.

**Methods:** This is a secondary analysis from a cluster randomized trial that embeds Healthy Eating and Active Living Taught at Home within Parents as Teachers (PAT). PAT is a national organization serving families prenatal through kindergarten, delivered by parent educators. After parent educators complete visits with mothers in the trial, they complete brief surveys including the question “Did issues with any of these come up during the visit?” with yes/no options for “Transportation,” “Housing,” “Food insecurity,” “Childcare,” “Financial constraint,” or “Other.”

**Results:** Among the 60 mothers with visit records in the months before and during (March–July 2020) COVID-19, 55% identified as Hispanic or Latino and 52% reported food insecurity at baseline. During COVID-19, financial constraints and other SDOH were as common as they were before COVID-19; childcare issues were discussed less frequently and food security was discussed more frequently. When comparing the number of SDOH parent educators reported discussing with mothers in visits that took place before COVID-19 with the number of SDOH discussed in visits during COVID-19, the number of SDOH increased for 41% for mothers identifying as Hispanic or Latino and only 8% for non-Hispanic or Latino mothers.

**Conclusions:** This study can help build an understanding of how COVID-19 is impacting families, and how these impacts may be inequitable.

Clinical Trial Registration Number: NCT03758638

## Introduction

The coronavirus pandemic, related social distancing, and the downstream economic effects of public health mitigation measures have highlighted critical issues related to health equity and the burden of chronic disease.^1^ Across the United States, the burden of COVID-19 and its severe complications have not been experienced equally. As of November 2020, individuals identifying as Hispanic and Latino are 1.7 times more likely to contract COVID-19, 4.1 times more likely to be hospitalized for COVID-19, and 2.8 times more likely to die from COVID-19 relative to those identifying as White, non-Hispanic.^2^ These inequities result in part from root causes, such as racism and systems of discrimination, which lead to a greater prevalence of underlying chronic conditions as well as increased exposure to the coronavirus owing to essential work demands, which limits social distancing opportunities.^3–5^ At the same time, with historic increases in unemployment, many economically vulnerable groups already at increased risk for chronic disease are experiencing challenging new stressors that warrant urgent attention. Families with low income, and particularly Hispanic and Latino families with low income, face increased pressure and stress related to health risks from COVID-19 and unmet needs (e.g., food and housing insecurity) because of the pandemic.^3–11^ This crisis has highlighted existing health disparities and threatens efforts to ensure everyone has fair and just opportunities to live their healthiest life. This heightens the importance of investigating how the pandemic is impacting and exacerbating obstacles to health.^12,13^

Families, and particularly Hispanic and Latino families, navigating the current crisis are particularly impacted by the changes in employment and childcare.^3–5,14,15^ Along with those changes have come new and increased resource needs (e.g., food, diapers and toilet paper, rent).^4,6^ Similar to the prevalence of the coronavirus and public health policy responses, these needs may differ geographically and intersect with the conditions in which people live, learn, work, and age (termed social determinants of health [SDOH]) for achieving and maintaining optimal health.^16^ Many organizations, such as Parents as Teachers (PAT), are helping families during this challenging time. PAT is a national home visiting organization serving families prenatal through kindergarten, and is delivered by parent educators, located in 1032 sites across all 50 states. The program trains parent educators to promote parental resilience, help families build social support, reach families where they are, and engage families who may not otherwise have access to such resources. Through an evidence-based model including home visits, group connections, screenings, and linkages to resources, PAT improves family and child well-being outcomes, reduces child neglect, and increases school readiness.^17^

Like many organizations in the context of a pandemic, PAT is finding new ways to reach, connect with, and provide support to the families they serve (e.g., using phone and video technology to conduct home visits).^18^ This is especially important, given home visiting programs may be impacted by the “digital divide”—a concept that refers to the inequality in access to technology that exists between communities owing to regional and demographic differences, particularly among socioeconomic groups. Of note, PAT has been testing and learning about virtual services delivery through interactive video conferencing since 2015, through a collaborative partnership with University of Southern California.^19^ Prior research has indicated that strategies for recruitment and enrollment in early childhood home visitation must be modified when applied to virtual home visitation as typically targeted families may not be able to envision the format of services being offered.^20^ To address the “digital divide,” PAT enabled visits during COVID-19 to be conducted by phone, when video conference technology is not available. The phone visits have a specific structure to follow per model guidance, yet additional research is needed to understand the impact of phone visits. PAT parent educators also drop off materials at participants' homes (e.g., handouts, materials for parent–child activities, diapers, or other resources), to support continued engagement. Whether virtual or in person, because of the personal nature of the relationship and the structure of the PAT program, many SDOH arise during conversations between the parent and the parent educator.

Much work has been carried out describing how adverse SDOH put individuals and communities at greater risk for acquiring and experiencing severe cases of COVID-19.^3,4,6,8,14,15,21^ In addition, the changes in unmet needs families face have been explored from multiple perspectives, for example by exploring requests for services from agencies responding to these requests through national surveys and qualitative interviews from families.^3,4,6,7,11,15,21^ National surveillance data from 211 helplines, which provide free information and referral services (e.g., financial, domestic, health, or disaster-related) by connecting people across North America to local resources,^22^ revealed a 486% increase in food-related requests during March 16–29, 2020.^7^ In addition, a survey from NPR, the Robert Wood Johnson Foundation, and the Harvard T.H. Chan School of Public Health found “a large majority of Latino households (72%) report facing serious financial problems during the coronavirus outbreak.”^15^ It is important to look beyond organizational reporting of request for services, as many households facing difficulty with SDOH report not seeking assistance.^15^ A description of the SDOH, which arise during visits with families before and after the start of the pandemic can add to this picture, providing a more complete understanding of how best to support families during this unprecedented time. This report describes the SDOH discussed during personal visits in the time leading up to the pandemic and then during the first 4 months of the pandemic from families across the United States.

## Methods

### Study design

These data are from an ongoing pragmatic cluster randomized controlled trial to evaluate dissemination and implementation of Healthy Eating and Active Living Taught at Home (HEALTH).^23^ The study evaluates the impact of HEALTH on healthy weight outcomes in overweight and obese mothers when it is implemented within PAT's existing infrastructure, assesses practice outcomes from the parent educator perspective, and determines the external validity of HEALTH.^23,24^ This study builds on a long history of collaborative work building healthy eating and activity content within PAT.^25–29^ Additional details about the study can be found elsewhere.^23^ In brief, parent educators at PAT sites randomized to the HEALTH arm receive training in the HEALTH curriculum; those at sites randomized to usual care will receive this training once their site has completed the trial. HEALTH is designed to be delivered over 24 months through evidence-based lifestyle change strategies to prevent weight gain and promote weight loss embedded within and delivered as part of PAT home visits. To be consistent with PAT's usual practice, HEALTH has a suggested but flexible visit structure, so the content, frequency, and number of visits are determined by the family's needs and preferences based on the parent educator's professional judgment. Personal visits as part of the study began in May 2019 and are ongoing, but for the current analysis, are included up to July 31, 2020, with those after March 13, 2020 (the date the of the National Emergency Declaration in the United States) considered to be during COVID-19.

### Participants and recruitment

Details about the study are included elsewhere,^29^ but a brief description is provided here. Owing to the pragmatic nature of the study, inclusion criteria were selected to mirror real-world PAT practice: female participants; 18–45 years of age; with a body mass index 25–45 kg/m^2^; not pregnant, <6 months postpartum, and not planning to become pregnant in the next 24 months; participating in or willing to participate in PAT; and able to give informed consent. Women were excluded if they were currently pregnant or planned to become pregnant in the next 24 months, unable to speak English or Spanish, planning to move out of the service area of the PAT site or to stop participating in PAT in the next 24 months, or unable to engage in a walking program. No specific racial or ethnic groups were targeted for recruitment. Although PAT sites are located in all 50 states, sites in eight states (i.e., Arizona, California, Delaware, Illinois, Kansas, New Jersey, Pennsylvania, and Tennessee) were participating in this study, to be included in the analysis. This study was approved by the Washington University in St. Louis Human Research Protection Office. All participants provided informed consent (Trial Registration: This study is registered at www.clinicaltrials.gov NCT03758638; First Posted: November 9, 2018; https://clinicaltrials.gov/ct2/show/NCT03758638).

#### Ethics approval and consent to participate

The Washington University in St. Louis Human Research Protection Office approved the study protocol and all participants provided verbal informed consent.

### Data collection and measurement

Measures for this study were drawn from visit records used to document adherence, quality of delivery, exposure to the intervention, and participant responsiveness or involvement. Parent educators in each arm (HEALTH and usual care) are asked to complete these visit records after each visit they complete with a mother enrolled in the study. The visit records, completed through online Research Electronic Data Capture (REDCap), surveys collect general data about the visit such as the date, the topic covered, the educators' perception of their ability to deliver the visit, and the mother's response to the visit. These records also asked educators: “Did issues with any of these come up during the visit?,” with yes/no options for “Transportation,” “Housing,” “Food insecurity,” “Childcare,” “Financial constraint,” or “Other.” The SDOH factors assessed in the visit records were guided by the Kaiser Family Foundation Social Determinants of Health framework^30^ including those most relevant to mothers with young children based on previous studies conducted in partnership with PAT. Parent educators are reimbursed $10 for their time completing each visit record.

At baseline, research staff measured participants' height and weight in accordance with National Health and Nutrition Examination Survey procedures.^31^ Sociodemographic measures (such as: age, marital status, number of children, and monthly income, food security^32^) were assessed by survey at baseline.

### Data analysis

All analyses were carried out using SAS version 9.1. Initial analyses explored whether there might be difference in baseline demographic characteristics between mothers included and those excluded (based on whether the mother received visits before and during COVID-19) in the current analysis; chi-squared tests were used to identify statistically significant differences (*p*<0.05). We then explored several ways of looking at the SDOH the parent educator reported discussing during the visit, using descriptive statistics. The first was to explore whether the number of SDOH decreased, increased, or stayed the same when comparing visits before and during COVID-19. We then looked at the number of SDOH the parent educator reported discussing in the visits before compared with the visits during SDOH as well as the difference (i.e., during minus before COVID-19). We present these stratified by participant ethnicity (i.e., Hispanic/Latino ethnicity, regardless of racial identification), as existing literature indicates an increased burden among Hispanic Americans.^3,4,29^

## Results

A total of 122 mothers had been enrolled in the trial as of July 31, 2020. Of these, 93 mothers had a visit record before COVID-19, and 60 had visit records before and during COVID-19. Table 1 provides the demographic characteristics combined for all the mothers enrolled in the study (*n*=122) as well as separated for those who had visits both before and during COVID-19 (*n*=60) and those who did not have a visit before and during COVID-19 (i.e., they either had only a visit before COVID-19, only a visit during COVID-19, or did not have a visit, *n*=62). The only differences between those two groups, which was statistically significant is in the mother's educational attainment and whether parent educators reported discussing housing issues during visits before COVID-19 (Table 1). Among those with visits in both time periods, 26% reported an income under $10,000, and 75% reported an income <$40,000; 52% of mothers reported food insecurity at baseline. Eighty-three percent of mothers reported receiving services from at least one program including Women, Infants, and Children, Supplemental Nutrition Assistance Program, and/or some other program at baseline. Most (55%) mothers identified as Hispanic or Latino, 28% of mothers identified as white/non-Hispanic or Latino, and 17% of mothers identified as non-Hispanic or Latino and a race other than white.

**Table 1.** Demographic Characteristics and Social Determinants of Health at Baseline of Mothers in the Study (*n*=122), and Separated for Those with a Visit Before and During COVID-19 (*n*=60) and Those That Did Not Have a Visit Before and During COVID-19 (*n*=62) from the Analysis

	Total (n=122)	Visit before and during (n=60)	No visit before/during (n=62)
	*n* (%)	*n* (%)	*n* (%)
Ethnicity			
Hispanic or Latino	60 (52.2)	32 (56.1)	28 (48.3)
Not Hispanic or Latino	55 (47.8)	25 (43.9)	30 (51.7)
Race/ethnicity			
Hispanic or Latino	60 (49.2)	32 (53.3)	28 (45.2)
White/not Hispanic or Latino	35 (28.7)	16 (26.7)	19 (30.7)
Black/not Hispanic or Latino	12 (9.8)	3 (5.0)	9 (14.5)
Asian/not Hispanic or Latino	4 (3.3)	4 (6.7)	0 (0)
Native Hawaiian or other Pacific Islander/not Hispanic or Latino	1 (0.8)	1 (1.7)	0 (0)
More than one race/not Hispanic or Latino	5 (4.1)	2 (3.3)	3 (4.8)
Race and ethnicity missing	5 (4.1)	2 (3.3)	3 (4.8)
Are you currently employed for wages/salary			
No	81 (66.4)	43 (71.7)	38 (61.3)
Yes	41 (33.6)	17 (28.3)	24 (38.7)
Income from all sources			
Under $10,000	28 (23.7)	15 (26.3)	13 (21.3)
$10,000–$19,999	25 (21.2)	13 (22.8)	12 (19.7)
$20,000–$29,999	25 (21.2)	10 (17.5)	15 (24.6)
$30,000–$39,999	13 (11.0)	5 (8.8)	8 (13.1)
$40,000+	27 (22.9)	14 (24.6)	13 (21.3)
Food security status			
Food secure	64 (53.3)	31 (52.5)	33 (54.1)
Food insecure	56 (46.7)	28 (47.5)	28 (45.9)
Number of children in home			
1	34 (27.9)	14 (23.3)	20 (32.3)
2	41 (33.6)	23 (38.3)	18 (29.0)
3	24 (19.7)	10 (16.7)	14 (22.6)
4+	23 (18.9)	13 (21.7)	10 (16.1)
BMI category			
Overweight (≥25)	48 (39.3)	26 (43.3)	22 (35.5)
Obese I (≥30)	36 (29.5)	17 (28.3)	19 (30.7)
Obese II (≥35)	20 (16.4)	10 (16.7)	10 (16.1)
Obese III (≥40)	18 (14.8)	7 (11.7)	11 (17.7)
Mom's education^a^			
Less/some than high school	27 (22.1)	19 (31.7)	8 (12.9)
High school graduate/GED/vocational school	45 (36.9)	17 (28.3)	28 (45.2)
Some college	25 (20.5)	10 (16.7)	15 (24.2)
College grad/Grad or professional	25 (20.5)	14 (23.3)	11 (17.7)
Do you receive help from any of the following programs (check all that apply)			
WIC	90 (73.8)	45 (75.0)	45 (72.6)
SNAP	56 (45.9)	28 (46.7)	28 (45.2)
Other	8 (6.6)	3 (5.0)	5 (8.1)
None	24 (19.7)	10 (16.7)	14 (22.6)
Sum of programs (i.e., WIC, SNAP, other)			
0	24 (19.7)	10 (16.7)	14 (22.6)
1	46 (37.7)	25 (41.7)	21 (33.9)
2	48 (39.3)	24 (40.0)	24 (38.7)
3	4 (3.3)	1 (1.7)	3 (4.8)
Did issues with any of these come up during the visit? Before COVID-19 (*n*=93)			
Transport	28 (30.1)	17 (28.3)	11 (33.3)
Housing	20 (21.5)	9 (15.0)	11 (33.3)
Food insecurity	12 (12.9)	6 (10.0)	6 (18.2)
Childcare	36 (38.7)	24 (40.0)	12 (36.4)
Financial constraint	38 (40.9)	23 (38.3)	15 (45.5)
Other	37 (39.8)	27 (45.0)	10 (30.3)
Sum of SDOH arising during visits, by parent educator report before COVID-19 (*n*=93)			
0	30 (32.3)	20 (33.3)	10 (30.3)
1	15 (16.1)	11 (18.3)	4 (12.1)
2	18 (19.4)	11 (18.3)	7 (21.2)
3	15 (16.1)	9 (15.0)	6 (18.2)
4	5 (5.4)	2 (3.3)	3 (9.1)
5	5 (5.4)	4 (6.7)	1 (3.0)
6	5 (5.4)	3 (5.0)	2 (6.1)

^a^ Difference between those included and excluded statistically significant.

BMI, body mass index; SDOH, social determinants of health; SNAP, Supplemental Nutrition Assistance Program; WIC, The Special Supplemental Nutrition Program for Women, Infants, and Children.

Of the 93 mothers with a visit before COVID-19, nearly one-third did not have a reported SDOH arise during the visits, but parent educators for 17% reported discussing four or more SDOH during visits before COVID-19 (bottom of Table 1). These results were similar for the 60 mothers with visits before and during COVID-19; 40% had reports of no SDOH discussed, whereas for 18% parent educators reported discussing four or more SDOH in the visits that took place during COVID-19.

Among mothers with visits before and during COVID-19, parent educators frequently reported discussing childcare (40%), financial constraints (38%), and other SDOH (e.g., addiction and recovery, employment; 45%) with mothers before COVID-19. During COVID-19, financial constraint (43%) and other SDOH (39%) remained common, but parent educators reported discussing childcare issues less frequently (28%). Food security, however, was discussed with only 10% mothers during pre-COVID-19 visits, but with 27% of mothers during COVID-19 visits (Table 2).

**Table 2.** Visit Before and During (*n*=60)

	Before COVID	During COVID	Change
	*n* (%)	*n* (%)	*n* (%)
Did issues with any of these come up during the visit?			
Transportation	17 (28.3)	11 (18.3)	
Housing	9 (15.0)	11 (18.3)	
Food insecurity	6 (10.0)	16 (26.7)	
Childcare	24 (40.0)	17 (28.3)	
Financial constraint	23 (38.3)	26 (43.3)	
Other	27 (45.0)	23 (38.3)	
Number of SDOH discussed during visits, by parent educator report			
0	20 (33.3)	24 (40.0)	
1	11 (18.3)	7 (11.7)	
2	11 (18.3)	12 (20.0)	
3	9 (15.0)	6 (10.0)	
4	2 (3.3)	4 (6.7)	
5	4 (6.7)	3 (5.0)	
6	3 (5.0)	4 (6.7)	
Diff in SDOH before/during COVID-19^a^			
−3			2 (3.3)
−2			6 (10.0)
−1			11 (18.3)
0			25 (41.7)
1			8 (13.3)
2			5 (8.3)
3			3 (5.0)
Difference in SDOH before to during COVID-19			
Decrease in SDOH			19 (31.7)
No change in SDOH			25 (41.7)
Increase in SDOH			16 (26.7)

^a^ Calculated as: SDOH during COVID-19 minus SDOH before COVID-19.

**FIG. 1.**
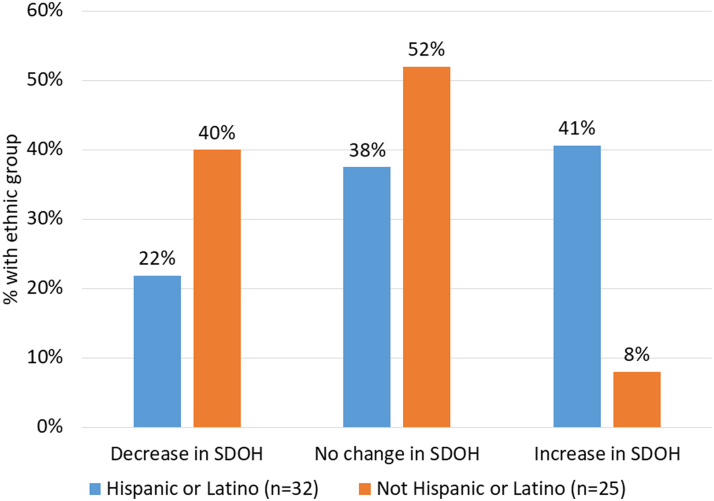
Difference in number of SDOH before relative to during COVID; ethnicity categories add to 100% (*n*=57). SDOH, social determinants of health.

When exploring the change in SDOH to decrease, stay the same, or increase from visits before to visits during COVID, Figure 1 provides the percent of mothers in each of these SDOH change categories, stratified by ethnicity. Parent educators reported a decrease in the number of SDOH discussed among non-Hispanic mothers during COVID-19 compared with before COVID-19; this was only the case for 22% of Hispanic mothers. Furthermore, whereas the number of SDOH parent educators reported discussing during visits increased in only 8% of non-Hispanic or Latino mothers, this increased in 41% of Hispanic or Latino mothers.

Cross-tabulating of the number of SDOH parent educators reported discussing during visits before COVID with those parent educators reported discussing during COVID, visualizes how the number of SDOH increased or decreased. In Table 3, the dark blue indicates fewer, the lighter blue indicates no change, and the lightest blue indicates an increase in the number of SDOH. Both panels show the percent of mothers in each category. Relative to the right panel, which includes mothers identifying as non-Hispanic and where only 8% of mothers are in light blue cells, the left panel, which includes mothers identifying as Hispanic or Latino, shows the distribution of mothers in many categories of increased SDOH. For example, parent educators reported two SDOH arising before COVID-19 and four arising during COVID-19 among 3.1% of Hispanic mothers, while this was the case for 0% of non-Hispanic mothers.

**Table 3.** Cross-Tabulating of the Number of Social Determinants of Health Parent Educators Reported Discussing During Visits Before COVID with Those During COVID-19

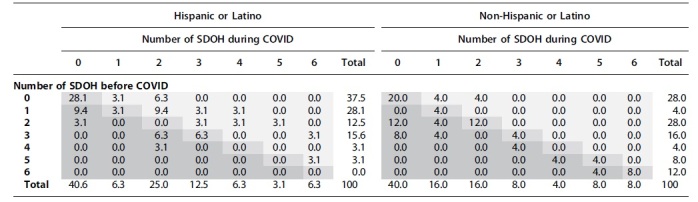

Each cell contains the percent of mothers in the category. Dark gray indicates fewer, the lighter gray indicates no change, and the lightest gray indicates an increase in the number of SDOH.

Comparing the change in the number of SDOH before to during COVID-19 (i.e., negative numbers indicate a decrease in the number of SDOH), Table 4 shows that for most mothers there was no or only a small change (defined as zero, increase or decrease of only 1) in the number of SDOH parent educators reported. However, whereas the parent educator for one (4%) non-Hispanic or Latino mother reported an increase of two or more SDOH from before to during COVID-19, and zero reported an increase of three SDOH, this was the case for four (12.5%) and three (9.4%) Hispanic or Latino mothers, respectively.

**Table 4.** Difference in Social Determinants of Health Before/During, *n* (%)

	Total	Ethnicity^a^	
Change		Hispanic or Latino	Non-Hispanic or Latino
−3	2 (3.5)	0	2 (8.0)
−2	6 (10.5)	2 (6.3)	4 (16.0)
−1	9 (15.8)	5 (15.6)	4 (16.0)
0	25 (43.9)	12 (37.5)	13 (52.0)
1	7 (12.3)	6 (18.8)	1 (4.0)
2	5 (8.8)	4 (12.5)	1 (4.0)
3	3 (5.3)	3 (9.4)	0
Total	57	32	25

Calculated as: SDOH during COVID-19 minus SDOH before COVID-19.

^a^ Hispanic/Latino ethnicity, regardless of racial identification; three participants did not report ethnicity.

## Discussion

This study explored the SDOH parent educators reported discussing during personal visits between mothers and parent educators, participating in a family-strengthening program, as reported by parent educators before and during COVID-19. This exploration identified that during COVID-19, financial constraint and other SDOH were common, as they were before COVID-19, but childcare issues were discussed less frequently and food security was discussed more frequently during COVID-19. In addition, whereas for many mothers, the number of SDOH parent educators reported discussing decreased or remained the same compared with visits before COVID-19, there were many increases in SDOH among mothers identifying as Hispanic or Latino.

The current findings align with national survey data, which has shown the prevalence of financial insecurity to be high. For example, among one sample of nonelderly adults, 31% reported they were unable to pay rent, mortgage, or utilities.^8^ Given school and daycare closures, it is notable that discussions of childcare issues were reported among fewer families during COVID-19 compared with before the pandemic, which may be because of job loss or furlough, allowing one or more caregivers to be at home to provide childcare. Furthermore, other work during this timeframe early in the pandemic documenting requests for assistance through 211 hotlines has demonstrated similar patterns in changes to the type of help requested, showing decreases in requests for childcare and transportation assistance.^33^ Similar to the current analysis, requests for food assistance from 211 increased dramatically during COVID-19. This study also aligns with work showing the economic impacts of COVID-19 are particularly challenging among Hispanic and Latino communities.^4,6^ Larger, national data suggest that pandemic-related job losses were experienced disproportionately by communities of color including those identifying as Hispanic or Latino,^4,5,9–11^ which may explain the increases of SDOH reported in the current analysis among mothers identifying as Hispanic or Latino. By exploring the topics discussed between families and their parent educators, this study provides another way of looking at how the pandemic is impacting Americans to augment survey and service system data collection.

A strength of this study is that it provides a unique view of the issues facing families before and during COVID-19, as it explores parent educator report of the SDOH they discuss with mothers. However, it is limited in that the study was not designed to explore SDOH during COVID-19; therefore, the SDOH categories were cruder than those used in other systems such as the 211 hotline or in surveys set up specifically to assess SDOH and are based on parent educator perception and report. The randomized trial was designed to evaluate the impact of an intervention on mothers' weight, relative to usual care PAT; however, in the current analysis, we did not stratify based on treatment arm. Furthermore, while the report is strengthened in that visits began before and continued during COVID-19, this analysis includes only participants for whom their parent educator had completed visit records both before and during COVID-19, the sample size is therefore limited and there is considerable variation in when each mother joined the study and began personal visits relative to the initiation of COVID-19 restrictions. The representatives of the participants is also limited; although we were able to explore differences by ethnicity, we were not able to present findings by race, which other studies have shown to be very important in COVID-19.^9^ All data about the SDOH were collected by parent educator self-report; therefore, these are subject to reporting error and bias.

### Health equity implications

This study provides a description of the SDOH parent educators reported discussing during personal visits with a diverse group of mothers participating in a family-strengthening intervention from across the United States. Such reporting can contribute to an understanding of how COVID-19 is impacting families; how these impacts may be inequitable, exacerbating existing disparities; and what services may be most need.

## Data Availability

The datasets for this article are not publicly available because of confidentiality requirements. Requests to access the datasets should be directed to Rachel Tabak (rtabak@wustl.edu).

## Abbreviations Used

BMI: body mass index
HEALTH: Healthy Eating and Active Living Taught at Home
PAT: Parents as Teachers
SDOH: social determinants of health
SNAP: Supplemental Nutrition Assistance Program
WIC: Special Supplemental Nutrition Program for Women, Infants, and Children
